# Author Correction: Mapping global acceptance and uptake of COVID-19 vaccination: a systematic review and meta-analysis

**DOI:** 10.1038/s43856-022-00190-9

**Published:** 2022-09-30

**Authors:** Qian Wang, Simeng Hu, Fanxing Du, Shujie Zang, Yuting Xing, Zhiqiang Qu, Xu Zhang, Leesa Lin, Zhiyuan Hou

**Affiliations:** 1 School of Public Health, Global Health Institute, Fudan University, Shanghai, China; 2 Department of Infectious Disease Epidemiology, London School of Hygiene & Tropical Medicine, London, UK; 3 Laboratory of Data Discovery for Health (D24H), Hong Kong Science Park, Hong Kong SAR, China

**Keywords:** Preventive medicine, Public health, Epidemiology, Epidemiology

Correction to: *Communications Medicine* 10.1038/s43856-022-00177-6, published online 12 September 2022.

There was a mistake in the Acknowledgements section of this article. The first sentence read ‘This work was funded by National Natural Science Foundation of China (No. 71874034), the Soft Science Research Project of Shanghai “Science and Technology Innovation Action Plan” (22692107600), and the National Institute for Health Research (EPIDZL9012) using UK aid from the UK Government to support global health research.’ However, it should have read ‘This research was commissioned by the National Institute of Health Research using Official Development Assistance (ODA) funding, (Grant ID: 16/137/109). The views expressed in this publication are those of the author(s) and not necessarily those of the NHS, the National Institute for Health Research or the Department of Health. This research was supported by National Natural Science Foundation of China (No. 71874034), and the Soft Science Research Project of Shanghai “Science and Technology Innovation Action Plan” (22692107600).’ This has now been corrected in the HTML and PDF versions of the article.

